# Extracellular Vesicles Contribute to Oxidized LDL-Induced Stromal Cell Proliferation in Benign Prostatic Hyperplasia

**DOI:** 10.3390/biology13100827

**Published:** 2024-10-16

**Authors:** Franco F. Roldán Gallardo, Daniel E. Martínez Piñerez, Kevin F. Reinarz Torrado, Gabriela A. Berg, Jael D. Herzfeld, Vanina G. Da Ros, Manuel López Seoane, Cristina A. Maldonado, Amado A. Quintar

**Affiliations:** 1 Centro de Microscopía Electrónica, Facultad de Ciencias Médicas, Universidad Nacional de Córdoba, Córdoba 5000, Argentina; franco.roldan.gallardo@unc.edu.ar (F.F.R.G.);; 2 Instituto de Investigaciones en Ciencias de la Salud (INICSA), Consejo Nacional de Investigaciones Científicas y Técnicas (CONICET), Córdoba 5000, Argentina; 3 Departamento de Bioquímica Clínica, Facultad de Farmacia y Bioquímica, Universidad de Buenos Aires, Ciudad Autónoma de Buenos Aires 1000, Argentina; 4 Instituto de Biología y Medicina Experimental (IBYME), Consejo Nacional de Investigaciones Científicas y Técnicas (CONICET), Ciudad Autónoma de Buenos Aires 1000, Argentina; 5 Sanatorio Allende, Sede Nueva Córdoba, Córdoba 5000, Argentina

**Keywords:** prostate hyperplasia, dyslipidemia, extracellular vesicles, metformin

## Abstract

**Simple Summary:**

Benign Prostatic Hyperplasia (BPH) is a prevalent condition among aging men, and recent research suggests it may be influenced by high cholesterol levels, especially oxidized LDL (OxLDL). However, how this happens is still not fully understood. This study aimed to explore how elevated OxLDL levels affect prostate cell growth and the release of small particles called extracellular vesicles (EVs), which cells use to communicate. By studying mice on a high-fat diet and human prostate cells exposed to OxLDL, we found that these conditions led to increased cell growth, potentially worsening BPH. We also discovered that OxLDL stimulated the production of EVs, further promoting cell proliferation. However, the drug metformin, commonly used to treat diabetes, was able to reduce this harmful growth. These findings provide new insights into how OxLDL may drive BPH progression and suggest that metformin could be a promising treatment to delay its development, potentially benefiting many men worldwide.

**Abstract:**

Background: Clinical and experimental evidence has linked Benign Prostatic Hyperplasia (BPH) with dyslipidemic and hypercholesterolemic conditions, though the underlying cellular mechanisms remain unclear. This study investigates the impact of dyslipidemia, specifically oxidized LDL (OxLDL), on prostatic stromal cell proliferation and the release of extracellular vesicles (EVs). Methods: Mice were fed a high-fat diet, and human prostatic stromal cells (HPSCs) were treated with OxLDL. Proliferation assays and EV characterization were performed to assess the role of EVs in BPH progression. Results: Pro-atherogenic conditions significantly increased cell proliferation in both murine prostatic cells and HPSCs. Treatment with metformin effectively inhibited OxLDL-induced proliferation. Additionally, OxLDL stimulated the production and release of pro-proliferative EVs by HPSCs, which further promoted cellular proliferation. Conclusions: The findings suggest that dyslipidemia drives prostatic stromal cell proliferation and EV secretion, contributing to BPH progression. Metformin demonstrates potential as a therapeutic agent to mitigate these effects, offering insight into novel strategies for BPH management. This study highlights the complex interaction between dyslipidemia, cell proliferation, and extracellular communication in the context of BPH pathogenesis.

## 1. Introduction

Benign Prostatic Hyperplasia (BPH) is a chronic and progressive condition, characterized by uncontrolled cellular proliferation in the transition zone of the prostate, which leads to gland enlargement and manifests as lower urinary tract symptoms (LUTSs) [1]. The prevalence of BPH is estimated to be around 50% in men over the age of 50, increasing by 10% for each decade of life [2]. Although several pharmacological therapeutic strategies are currently available, for most patients, partial or radical prostatectomy and its associated complications become inevitable [3].

Changes in lifestyle and dietary habits, along with the rise of the “Western Diet” in recent decades, have sparked concerns regarding the role of dyslipidemia in the pathophysiology of diverse conditions in many systems and organs [4]. In fact, hyperlipidemia/dyslipidemia constitutes a primary risk factor for atherosclerosis and cardiovascular disease due to a spectrum of interconnected metabolic and immune disturbances that culminate in systemic inflammation and oxidative stress, among other processes [5,6]. Although it has been suggested that such a context may have a potential pathogenic role in the abnormal growth of the prostate [7,8], the precise cellular and molecular mechanisms underlying dyslipidemia-induced cellular proliferation remain unclear. Hypercholesterolemia often results in the accumulation of the low-density lipoprotein (LDL) in the subendothelial tissue, where it is proposed to undergo modifications as oxidation, leading to the formation of oxidized LDLs (OxLDL). This process alters the structure and functionality of LDL [9], being a crucial step for lipid accumulation and vascular inflammation in atherosclerosis and other vascular pathologies. Additionally, it has been reported that OxLDL can trigger cellular proliferation in several tissues both in vitro and in vivo [10,11]. For instance, in endothelial cells, it promotes proliferation by activating the Rho/Akt signaling pathway [11] and modulates cell cycle and cell survival in vascular cells by regulating p27Kip1 via Rho [12] and in smooth muscle cells [13]. In the prostate, OxLDL has been reported to induce the secretion of proinflammatory cytokines [14], likely impacting inflammatory and proliferative processes in the gland.

Extracellular vesicles (EVs) have emerged as crucial mediators of intercellular communication in various physiological and pathological conditions due to the diverse biomolecules they are able to transport [15]. Strikingly, EVs can drastically change cellular phenotypes and behaviors such as proliferation, migration, invasion and apoptosis [16,17,18,19]. In the context of dyslipidemia, OxLDL has been shown to promote EV release by endothelial cells, regulating inflammation during atherosclerosis through cargoes such as miR-155 [20]. In the same line of evidence, OxLDL-pulsed, macrophage-derived EVs have been demonstrated to induce cell migration in atherosclerotic plaque [21]. Moreover, OxLDL can stimulate platelets to secrete EVs with procoagulant effects that amplify oxidative stress in proatherogenic scenarios [22]. Considering these diverse roles in cellular communication during dyslipidemia, we hypothesized that EVs might also be involved in the alleged negative actions of OxLDL on prostatic cells [14,23]. Therefore, we analyzed the effects of a dyslipidemic environment on mouse prostate proliferation and on human prostatic stromal cells (HPSCs) derived from BPH patients and examined the involvement of EVs.

## 2. Materials and Methods

### 2.1. Mice and High-Fat Diet (HFD)

Male C57BL/6 mice, aged 4 weeks, were housed at IBYME-CONICET and INICSA CONICET (Argentina) and fed either a standard chow diet (6% fat, 24% protein, 40.5% carbohydrates, 3210 kcal/kg) or a HFD (30.3% fat, 17.8% protein, 30% carbohydrates, 4640 kcal/kg) for 12 weeks [24]. The mice were kept under a controlled photoperiod (14 h light/10 h darkness) with ad libitum access to water. All procedures adhered to the Guide for the Care and Use of Laboratory Animals published by the National Institutes of Health (NIH) and the approval for the animal use protocol was obtained from the Institutional Animal Care and Use Committee (IACUC) of IBYME-CONICET Argentina (code 22_2017). At the end of the protocol, blood was collected for total cholesterol determination; prostatic complexes were extracted, fixed with 4% formaldehyde, embedded in paraffin, and sectioned into 4 µm histological sections for routine hematoxylin–eosin staining and immunocytochemistry for the proliferation marker Ki-67.

### 2.2. Primary Cultures of Prostatic Stromal Cells and Cell Lines

Prostate tissue samples were collected from BPH-diagnosed patients aged 50 to 70 years via TURP and placed in MCDB131 medium (Sigma-Aldrich, St. Louis, MO, USA) on ice. Hypertension was accepted as a comorbidity, but not diabetes or conditions such as hypercholesterolemia. Mechanical and enzymatic digestion using type IA collagenase (200 U/mL, Sigma-Aldrich) was performed for 1.5 h. Cells were then seeded into 6-well culture plates in MCDB131 medium supplemented with 15% fetal bovine serum (Internegocios S.A., Buenos Aires, Argentina). Cultures were maintained for a minimum of 15 days and underwent at least 6 passages to ensure stromal purity before experimental protocols [25]. The study was approved by the Bioethics Committee of Sanatorio Allende (Córdoba, Argentina).

Prior to OxLDL stimulation, HPSCs were kept in serum-free medium for 24 h. LDL was obtained from human plasma and underwent oxidation with Cu^2+^ as previously described [26]. Two types of OxLDL samples were obtained: moderately oxidized (OxLDL1) and highly oxidized (OxLDL2). The difference between OxLDL1 and OxLDL2 molecules is based on their degree of oxidation, determined by their exposure to Cu^2+^. For OxLDL1, LDL was exposed to Cu^2+^ for 4 h at 37 °C, followed by the addition of 2 mmol/L EDTA to halt the process. Conversely, the oxidation process for OxLDL2 was never completely stopped because EDTA was not added after the 4 h exposure to Cu^2+^ [26]. HPSCs were stimulated with OxLDL at concentrations of 5, 20, and 100 µg/mL to simulate a dyslipidemic environment. Metformin (2 and 10 mM), atorvastatin (20 µM and 2.5 µM), or a combination of both at high and low concentrations were then used as co-stimuli for 24 h to assess their potential positive effects on cell proliferation within this dyslipidemic context [26].

As these drugs are commonly used to effectively treat MS components. The human monocytic THP-1 cell line (from ATCC) was cultured in RPMI1640 medium (Thermo Fisher Scientific Inc., Waltham, MA, USA) supplemented with 10% FBS and stimulated with OxLDL1 and OxLDL2 at a concentration of 20 µg/mL.

### 2.3. Proliferation Assays

Cell proliferation and viability were assessed using the BrdU incorporation assay, Ki-67 immunocytochemistry, and resazurin test. Briefly, cells were seeded on coverslips in 24-well culture plates, with 10 µM 5-Bromo-2′-Deoxyuridine (Sigma-Aldrich, St. Louis, MO, USA) being added for the last 6 h of treatments. Cells and prostatic tissues were fixed in 4% formaldehyde and subjected to antigen retrieval using 0.1 M citrate buffer, followed by permeabilization using 0.25% Triton X-100. Coverslips were then incubated with 1 U/µL DNase (1/10, Zymo Research, Irvine, CA, USA) diluted in Krebs buffer (Sigma-Aldrich) and subsequently with primary antibodies (1/30 Ki-67 Cat. 550609 and 1/200 Anti BrdU Cat. 555627, both in 1% BSA, BD Pharmingen, San Diego, CA, USA) overnight. An Alexa594-conjugated anti-mouse antibody (Invitrogen, Carlsbad, CA, USA) was used for 1 h for BrdU detection and DAPI for nuclear staining. For Ki-67, the Avidin-Biotin peroxidase Complex (ABC, Thermo Fisher Scientific Inc.) was added and the immunoreactivity was visualized using DAB (Sigma-Aldrich). In both techniques, 1000 cells were counted to determine the percentage of proliferating cells. For THP-1 cells growing in suspension, proliferation was evaluated by counting the total number of cells using a Neubauer Chamber. Cell viability and metabolic activity of both HPSCs and THP-1 cells were assessed by adding Resazurin (1 µg/mL, Thermo Fisher Scientific Inc.) to treatments in 96-well plates. Absorbance was then quantified at 560 nm using a GloMax^®^ Multi Detection System spectrophotometer (Promega, Madison, WI, USA).

### 2.4. Isolation of HPSCs-Derived EVs

HPSCs were cultured in 100 mm diameter adherent plates; upon reaching 80% confluence, the culture medium was replaced with serum-free MCDB131 for a 24 h period followed by 20 µg/mL OxLDL1 treatment for 24 h. The medium was then replaced with serum-free MCDB131 for another 24 h period to allow the accumulation of EVs in the medium. Supernatants were then collected, filtered through sterile filters with a pore size of 0.22 µm, and underwent ultracentrifugation to isolate EVs according to a standardized protocol [27] and the recommendations of the MISEV guidelines [28]. Briefly, conditioned media were subjected to 10 min at 200 g for 3 cycles (G146D centrifuge, Gelec^®^, Ciudad Autónoma de Buenos Aires, Argentina) and 20 min at 2000× *g* (Allegra 64R Centrifuge, Beckman Coulter, Brea, CA, USA) to remove cellular debris and apoptotic bodies, respectively. Finally, ultracentrifugation was performed at 150,000× *g* for 50 min (Optima MAX-TL ultracentrifuge, Beckman Coulter) to obtain a pool of EVs comprising both microvesicles and exosomes. The EVs were then resuspended in 15 µL of 1× PBS and stored at −80 °C until further analysis.

### 2.5. Cell and EV Characterization by Transmission Electron Microscopy (TEM)

Cells were fixed in Karnovsky solution for 15 min. A post fixation step was performed using 1% osmium tetroxide for 1 h and then, the samples were dehydrated in 50%, 70%, 90%, and 100% acetone for 15 min at each step. Afterwards, they were embedded in EPON Araldite resin (Electron Microscopy Sciences, Hatfield, PA, USA) and allowed to polymerize at 60 °C for 48 h. Thin sections (90 nm) were prepared using a diamond blade on a JEOL LTD JUM-7 ultramicrotome, contrasted with uranyl acetate and lead citrate before being observed under a Zeiss LEO 906E transmission electron microscope.

For EVs, 5 µL of PBS containing EVs was fixed in 2% formaldehyde for 2 h and then placed onto 200-mesh nickel grids coated with Formvar. To prevent non-specific binding, the grids were incubated in 5% BSA for 10 min. Monoclonal primary antibody CD63 (1 µg/mL, Santa Cruz Biotechnology, Dallas, TX, USA) was added for 1 h. Protein A conjugated with colloidal gold (1/30, Electron Microscopy Sciences) was added for 30 min. For counterstaining, the EVs were exposed to uranyl-oxalate, followed by immersion in methylcellulose-AU for 10 min. After air-drying, they were visualized via TEM, digitally recorded and quantified. Fifty micrographs were systematically taken for each TEM grid. The number and size of EVs were counted and recorded using the Line Tool in the ImageJ software v1.52a. Subsequently, extrapolation was performed considering the analyzed area and the total area of the grid versus the volume of EVs in µL used in order to obtain the number of EVs/µL for each treatment and size range.

### 2.6. EV-Induced Cell Proliferation

EVs were isolated from two sources: HPSCs controls and HPSCs stimulated by 20 µg/mL OxLDL1 for 24 h. These EVs were normalized according to the number of secreting cells (250,000), resuspended in 50 µL of PBS and used as stimuli in experiments conducted in 24-well plates over 24 h. Experiments involved both PC3 cell line epithelial cells and HPSCs, cultured according to standard protocols [29,30]. HPSCs were used to evaluate the effect of EVs on the induced proliferation of prostatic stromal cells, while the PC3 cell line was used to study this effect on epithelial compartment cells, thus evaluating the two main components of the prostate gland. Notably, primary cultures from which the EVs were extracted served as both EV sources and recipient cells, enabling an analysis of the EV effects on stromal cells from the same individual. The impact of EVs on cell proliferation was also examined through Ki-67 immunocytochemistry as detailed previously.

### 2.7. Statistical Analysis

Student’s *t*-test and analysis of variance (ANOVA) were conducted, followed by Tukey’s post hoc comparisons when necessary. To denote statistically significant differences between means, the following reference system was adopted: (*) for *p* < 0.05, (**) for *p* < 0.01, or (***) for *p* < 0.001. GraphPad Prism 9.4 software was employed for performing the statistical tests and generating graphical representations of the data. All experiments were performed at least three times, each in triplicate.

## 3. Results

### 3.1. Mice on a HFD Displayed an Increase in Prostate Cell Proliferation

**Figure 1:**
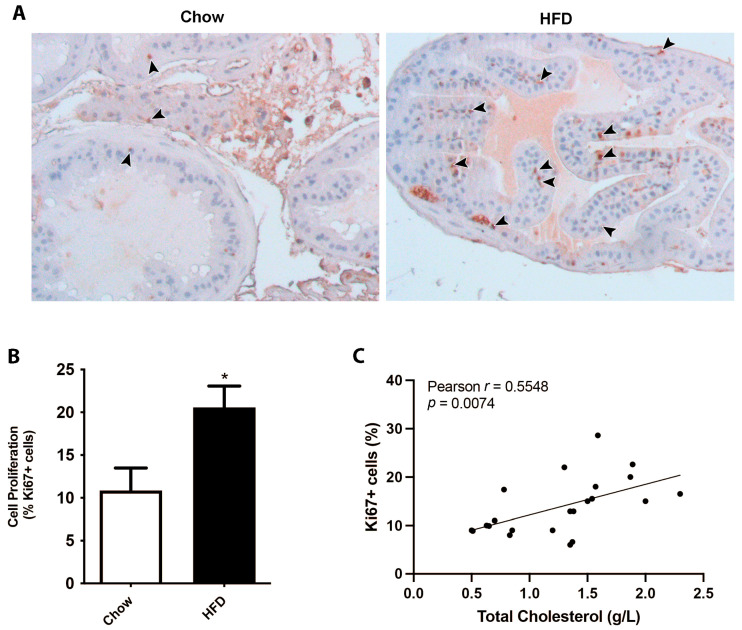
Hypercholesterolemia induced by a high-fat diet promotes cell proliferation in the prostate gland. Mice were fed on a HFD for 12 weeks and their prostates were processed and analyzed by Ki-67 immunohistochemistry. (**A**) Representative images of prostate glands immunostained for the proliferation marker Ki-67 showing an increase in HFD-fed mice (brown nuclei, arrowheads). (**B**) Quantification of Ki-67-positive stromal cells in ventral prostates from chow- or HFD-fed mice (mean ± SEM; *n* = 10 per group; * *p* < 0.05). (**C**) Correlative analysis showing serum cholesterol levels and prostatic Ki-67 positive counts (*n* = 20, Pearson’s correlation test).

In order to analyze the consequences of dyslipidemic scenarios on prostatic growth, we utilized a model of hypercholesteremia by feeding mice a HFD and then examining the prostate complex, compared to a chow diet. Although there were not significant differences in prostate weight or volume, the immunohistochemistry of the proliferative marker Ki-67 revealed a significant two-fold increase in the number of positive cells in mice fed a HFD in ventral prostate sections. Both stromal and epithelial compartments showed increased prostatic cell proliferation in the HFD group, which was positively correlated with circulating total cholesterol levels in mice (Figure 1). These findings support the hypothesis that dyslipidemic conditions have a detrimental effect on the prostate, promoting cellular proliferation.

### 3.2. OxLDL Promoted Proliferation and Phenotypic Changes in Prostatic Stromal Cells Derived from Patients with BPH

**Figure 2:**
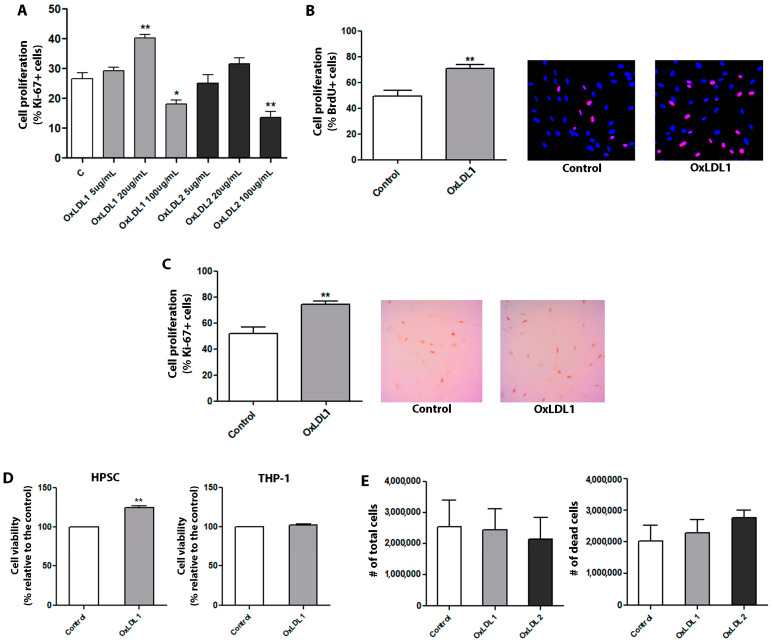
OxLDL at 20 µg/mL increases cell proliferation and viability of HPSCs. (**A**) HPSCs from patient HPSC-1 were used for the initial proliferation assay. Ki-67 immunocytochemistry analysis was performed to evaluate the effects of two types of OxLDL: moderately oxidized (OxLDL1) and highly oxidized (OxLDL2). Although OxLDL1 at 20 and 100 µg/mL and OxLDL2 at 100 µg/mL showed changes in cell proliferation (** *p* < 0.01, * *p* < 0.05, and ** *p* < 0.01 vs. control, respectively), OxLDL at 20 µg/mL was the only concentration without signs of cell toxicity. Additionally, OxLDL at 20 µg/mL induced an increase in cell proliferation (** *p* < 0.01 vs. control). (**B**) Quantification and representative merged images of DAPI/BrdU showing the proliferative effects of OxLDL1 at 20 µg/mL for 24 h vs. control (** *p* < 0.01), which were evaluated in different primary cultures of HPSCs by BrdU incorporation (*n* = 7). (**C**) The same proliferative effect of OxLDL1 at 20 µg/mL for 24 h vs. control (** *p* < 0.01) was evaluated in primary cultures of HPSCs by Ki-67 assay (*n* = 8). (**D**) Resazurin absorbance assay for HPSCs and THP-1 stimulated with OxLDL1 at 20 µg/mL for 24 h was carried out to assess cell viability. OxLDL1 at 20 µg/mL increased cell viability compared to control (** *p* < 0.01). (**E**) Assays with OxLDL1 and OxLDL2 stimuli in THP-1 cells evaluating the number of total live and apoptotic cells. Neither OxLDL1 nor OxLDL2 exerted changes in the cell number of THP-1 cells. All tests were performed in two replicates, each in triplicate. Error bars represent mean ± SEM.

BPH is the most frequent condition associated with pathological growth of the prostate [2] and it has been suggested to be sustained by dyslipidemia/hypercholesterolemia [8]. Considering that the oxidized form of the LDL is a key pathogenic effector in dyslipidemic contexts [9], we aimed to evaluate how prostatic cells from BPH patients respond to OxLDL To this end, we first established primary cultures of stromal cells from eight patients, identified as HPSC-1, HPSC-2, HPSC-3, HPSC 4, HPSC-5, HPSC 6, HPSC-7, and HPSC-8. Following 15 days of growth, these HPSCs exhibited a spindle-shaped morphology and a well-developed cytoskeleton; ultrastructural and histochemical analyses demonstrated a myofibroblastic profile in these primary HPSCs (Figure S1). The effects of OxLDL1 (moderately oxidized) and OxLDL2 (highly oxidized) on HPSCs were then compared at several concentrations. As shown in Figure 2A, 100 µg/mL OxLDL exhibited substantial toxicity for both types, resulting in low cell viability and reduced proliferation. On the other hand, 5 µg/mL of both did not induce significant changes in cell viability, apoptosis, and proliferation compared to controls. When stimulated with a 20 µg/mL concentration, HPSCs showed a significant increase in cell proliferation in response to both OxLDL1 and OxLDL2 compared to controls. However, this concentration also led to elevated cell death for OxLDL2, as observed in primary cultures during the treatment phase (Figure 2A). Based on these findings, 20 µg/mL of OxLDL1, the moderately oxidized form, was selected for all subsequent experiments. Accordingly, HPSCs stimulated by OxLDL1 for 24 h exhibited an increase in cell viability and cell proliferation, as determined by resazurin absorbance and BrdU assay (Figure 2B), as well as by Ki-67 immunocytochemistry (Figure 2C) in the eight primary cultures. To check the specificity of these effects in cell proliferation, the monocytic THP-1 cell line, which is typically used for studying LDL effects, was treated with OxLDL1 and OxLDL2 at a concentration of 20 µg/mL for 24 h. Despite being uptaken by THP 1 cells (Figure S2), OxLDL did not promote significant changes in cell viability (Figure 2D) or proliferation (Figure 2E) in this cell line.

**Figure 3:**
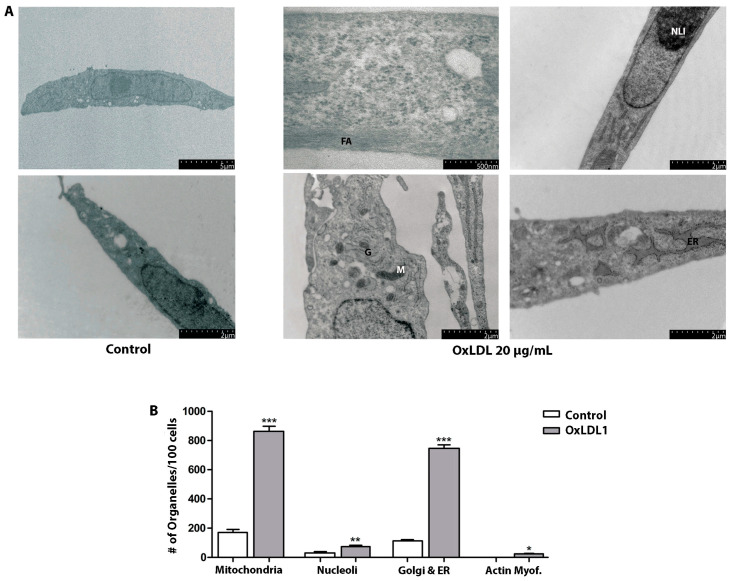
OxLDL causes morphological and ultrastructural changes in HPSCs. (**A**) Ultrastructure of HPSCs derived from patient HPSC-1, treated with OxLDL1 at 20 µg/mL for 24 h, and evaluated by TEM. Cells stimulated by OxLDL display frequent actin filaments (FAs), dilated endoplasmic reticulum (ER), numerous mitochondria (M), Golgi apparatus (G), and prominent nucleoli (NLI). (**B**) Quantification of organelles observed by TEM for HPSCs from patient HPSC-1, control vs. treated with OxLDL1 at 20 µg/mL for 24 h. There was a significant increase in the number of mitochondria (*** *p* < 0.001), nucleoli (** *p* < 0.01), Golgi and endoplasmic reticulum (*** *p* < 0.001), and actin myofibrils (* *p* < 0.05) when stimulated by OxLDL1 compared to the control. All tests were performed in two replicates, each in triplicate. Bars represent mean ± SEM.

At the ultrastructural level, control HPSCs exhibited a myofibroblastic phenotype, which included the presence of prominent rough endoplasmic reticulum and Golgi apparatus, abundant free ribosomes, signs of secretion granules, and peripherally located microfilament bundles. The OxLDL treatment resulted in markedly ultrastructural alterations (Figure 3A), along with an increase in the number of organelles associated with cellular metabolism and contractile activity (Figure 3B). Evidence of cellular stimulation after OxLDL included the occurrence of numerous nucleoli, an increment in proteinopoietic organelles, myofilaments with focal densities, and scarce fibronexus. External fibronectin fibrils were rarely observed. These findings align strongly with increased cell proliferation after OxLDL stimulation, indicating that OxLDL and dislipidemic factors promote a more aggressive phenotype in prostatic stromal cells in the context of BPH.

**Figure 4:**
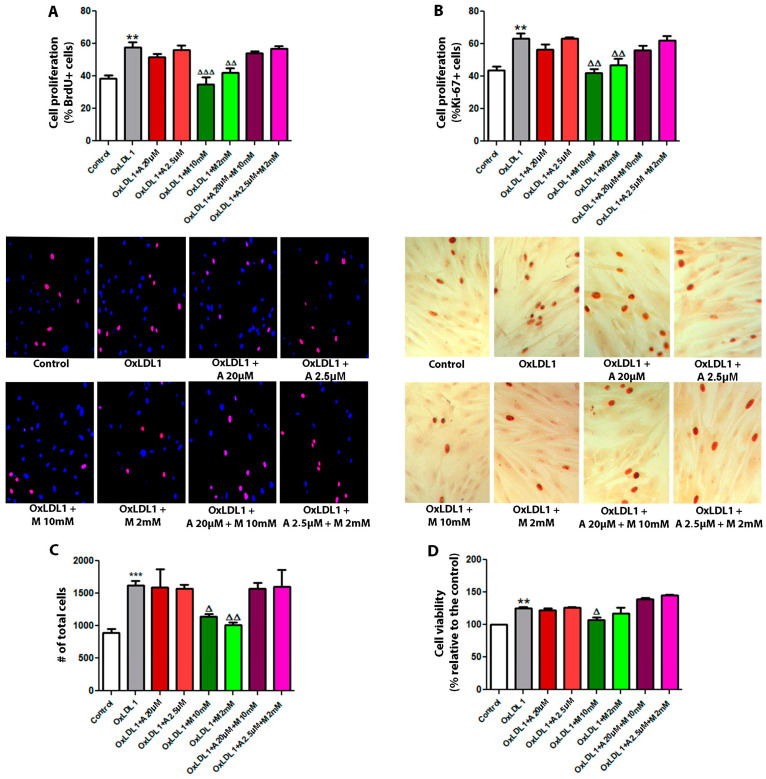
Metformin inhibits the proliferative effect of OxLDL on HPSCs. Cells were stimulated with 20 µg/mL of OxLDL1, and atorvastatin, metformin, or a combination of both were used as co-stimuli, each at low and high doses for 24 h. (**A**) Cell proliferation assays by BrdU incorporation were carried out in all primary cultures of HPSCs. Images of cells are representative of patient HPSC-6. OxLDL1 enhanced cell proliferation compared to the control (** *p* < 0.01), while metformin inhibited the proliferative effect induced by OxLDL1 (^ΔΔ^ *p* < 0.01 and ^ΔΔΔ^ *p* < 0.001 vs. OxLDL1). (**B**) A similar effect was observed by Ki-67 immunocytochemistry analysis on HPSCs. Representative images of cells are from the same patient HPSC-6 (** *p* < 0.01 for OxLDL1 vs. C, and ^ΔΔ^ *p* < 0.01 for metformin vs. OxLDL1). (**C**) Determination of the total cell number was performed on cells from patient HPSC-5, HPSC-6 and HPSC-7. OxLDL1 increased the cell number compared to the control (*** *p* < 0.001), while metformin reduced the cell number compared to OxLDL1 (^Δ^ *p* < 0.05 and ^ΔΔ^ *p* < 0.01). (**D**) Cell viability assay evaluated by resazurin absorbance was performed on cells from patient HPSC-5, HPSC-6 and HPSC-7. All the tests were performed in two replicates, each in triplicate. Bars represent mean ± SEM.

In the management of dyslipidemia and metabolic disorders, lowering LDL levels is the main strategy for reducing cardiovascular risk. Statins, such as atorvastatin, are the first-line therapy, followed by certain glucose-lowering drugs such as metformin, which has been demonstrated to have additional benefits [31]. Interestingly, atorvastatin and metformin also have direct effects on cellular proliferation [32] and we wondered if these drugs might be able to mitigate OxLDL-induced HPSCs proliferation. Treatment of HPSCs with metformin at both doses (2 and 10 mM) abrogated OxLDL-induced cell proliferation and viability, as determined by BrdU incorporation, Ki-67 immunocytochemistry, total cell count, and Resazurin assays (Figure 4). Combination with atorvastatin did not result in a reduction in OxLDL-induced cell proliferation. Moreover, atorvastatin alone did not exhibit inhibitory effects on cell proliferation. This finding was consistent across all primary cultures evaluated, which suggests a potential secondary antiproliferative role for metformin, beyond its hypoglycemic effects, in attenuating the proliferative processes in BPH under dyslipidemic conditions.

### 3.3. HPSCs Secreted EVs with Proliferative Activity in Response to OxLDL Stimulation

**Figure 5:**
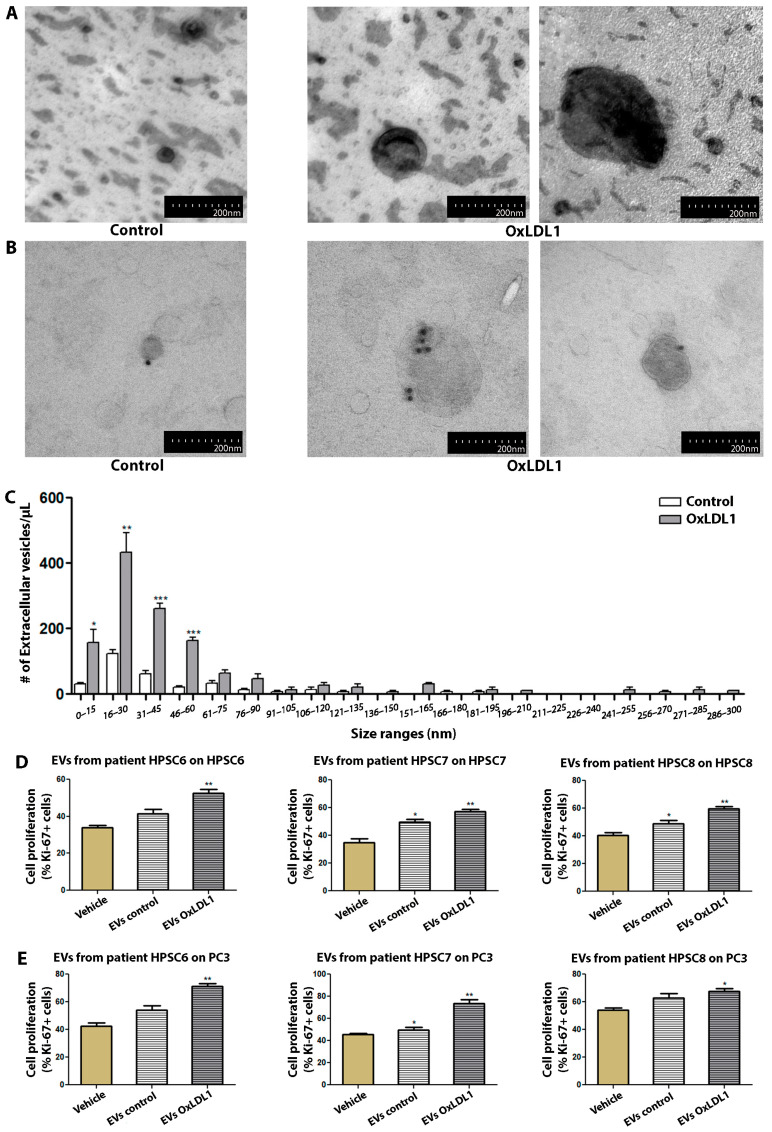
EVs derived from OxLDL-stimulated HPSCs exert a pro-proliferative effect on prostatic cells. (**A**) Images of HPSCs-derived EVs from patient HPSC-4, treated with OxLDL1 at 20 µg/mL for 24 h, isolated by ultracentrifugation and visualized by TEM. (**B**) Immunolabeling for CD63 in EVs derived from patient HPSC-4, using colloidal gold particles. (**C**) The histogram of size range distribution of EVs derived from HPSCs of patient HPSC-4 shows an increase in the number of EVs, mainly in the smaller size ranges (* *p* < 0.05, ** *p* < 0.01 and *** *p* < 0.001 vs. control). EVs obtained from HPSCs stimulated with OxLDL1 at 20 µg/mL for 24 h (EVs OxLDL1) and the control (EVs control) were used as stimuli on HPSCs from the same patient (**D**) and on prostatic epithelial cells from the PC3 line (**E**). EVs from OxLDL-stimulated HPSCs induced a significant proliferative effect on HPSCs and PC3 cells in all assays (** *p* < 0.01 and * *p* < 0.05 vs. vehicle). The proliferative effect was evaluated by Ki-67 and all tests were performed in three replicates, each in triplicate. Bars represent mean ± SEM.

As EVs have emerged as a central player in cell communication, supporting multiple cellular processes such as migration, survival, and proliferation in different systems, we wondered whether HPSCs are able to release EVs. The electron microscopic analysis of EVs isolated by ultracentrifugation from control and OxLDL-stimulated HPSCs revealed mainly concave, calyx-shaped structures (Figure 5A), with a peak size range of 15–30 nm structures (small EVs, previously recognized as exosomes). Immunogold staining for the tetraspanin CD63 also confirmed the identity of the EVs (Figure 5B). OxLDL induced a significant increase in the number of smaller EVs, particularly in the 15 to 60 nm range (Figure 5C).

To address the function of these EVs, supernatants from vehicle- and OxLDL-treated cells of three different BPH patients were subjected to ultracentrifugation, with the resulting EVs being used to stimulate HPSCs or PC3 cells (Figure 5D,E). OxLDL-induced EVs significantly increased cell proliferation compared to the vehicle- or control EV-treated HPSCs. Interestingly, OxLDL-pulsed EVs showed a similar proliferative activity on the PC3 epithelial cell line, suggesting that EVs can promote cell proliferation in both autocrine and paracrine manners in the context of BPH.

## 4. Discussion

The relationship between dyslipidemia and abnormal prostatic growth has been widely studied over the last decade. Although compelling evidence exists from epidemiological studies, limited information is still available on the cellular mechanisms explaining such a correlation. Our study found that HFD, which elevates LDL levels, induces prostatic cell proliferation in vivo, as has also been reported by another group [33]. Additionally, primary cultures of HPSCs from patients with BPH showed increased proliferation in response to OxLDL, which was inhibited by metformin. Furthermore, OxLDL augmented the secretion of pro-proliferative EVs by HPSCs, suggesting a potential mechanism underlying BPH progression in pro-atherogenic and dyslipidemic conditions.

LDL and its oxidized forms are known to cause vascular and microcirculatory lesions in various tissues in the contexts of dyslipidemia, MS, chronic inflammation, and hormonal imbalance [14]. In vitro, OxLDL can induce proliferation in human endothelial cells [34,35] as well as in prostatic PC3 and LNCaP cell lines [36], and has been implicated in promoting angiogenesis in C4-2 cells through LOX-1 receptor activation [36]. Moreover, Haga et al. have linked OxLDL to prostatic growth, attributing it to atherosclerosis-induced chronic inflammation via LOX-1 [37]. Our ultrastructural observations suggest an increment in both metabolic function and contractile activity of HPSCs after OxLDL, which promotes features of aggressive myofibroblasts [38,39]. Similar changes have been reported in cardiac myofibroblasts exposed to OxLDL, exacerbating a pro fibrotic phenotype [40,41,42]. Our findings underscore the pathological role of OxLDL in BPH and support the hypothesis linking dyslipidemia, inflammation and MS components with benign prostatic growth and LUTS [14].

Metformin and atorvastatin are widely prescribed for pro-atherogenic conditions like dyslipidemia, diabetes, and MS [31]. Notably, these drugs have also shown potential in regulating cell proliferation in different contexts [43,44,45]. Our results demonstrated antiproliferative effects for metformin, while atorvastatin did not impact OxLDL-induced proliferation of HPSCs in vitro. However, the combination of metformin and atorvastatin failed to counteract cell proliferation, indicating that intricate signaling pathways are involved. Different anti-proliferative mechanisms of action for metformin have been reported, including mTOR inhibition, AMPK activation, modulation of protein synthesis and cell growth, and the activation of p53 [46]. This discovery offers insight into metformin’s effects and the rationale for potential application in patients with BPH.

Over the last few years, studies have demonstrated that dyslipidemic states can increase the secretion of EVs by various cell types, including macrophages [21], endothelial cells [20], and platelets [22]. Furthermore, OxLDL-induced EV secretion appears to be mediated by the activation of scavenger receptors LOX-1 and CD-36 [47]. Here, we demonstrate for the first time that OxLDL stimulates the production and secretion of pro-proliferative EVs by HPSCs from patients with BPH. Previous studies have shown that prostatic tumor cells from the PC3 and LNCaP lines, as well as the WPMY-1 cell line, secrete EVs [48,49,50,51]. Additionally, EVs have been isolated from primary cultures of prostate cancer patients and healthy individuals, but not from patients with BPH [52]. The presence of EVs in prostatic histological sections from patients with cancer and BPH has also been compared by TEM, but without being able to distinguish between different cell types [53]. However, it remains uncertain whether these EVs share morphological and functional features with classical prostasomes or other microparticles secreted by the prostate gland [49]. Likewise, our findings confirm and provide conclusive evidence of the ability of prostatic stromal cells from BPH patients to release EVs with pro-proliferative capacities. Our findings are significant in this underexplored area, highlighting prostatic stromal cells as major contributors to the pathology and modulators of the extracellular milieu. In fact, the interactions between stromal and epithelial cells control the strict balance in pro- and anti-proliferative signals, which is dysregulated in BPH. In this scenario, autocrine and stroma-to-epithelium communications mediated by EVs appear to be an important player in the proliferative process promoted by dyslipidemia. This effect can be ascribed to the multiple EVs cargoes; for instance, miR-92a-3p, which inhibits the expression of thrombospondin 1, has been shown to regulate EV-induced cellular proliferation in the context of OxLDL challenges [50].

The observations of the present work could be strengthened by analyzing in vivo the therapeutic effect of metformin on pro-proliferative EVs secreted by prostatic cells in dyslipidemic contexts. In addition, studying the cargo of EVs in BPH and how they are regulated in that microenvironment would greatly enhance these findings.

## 5. Conclusions

In summary, this study provides strong evidence that dyslipidemia has a significant pathogenic impact on prostate cell proliferation and phenotype, both in animal models and in human stromal cells derived from patients with BPH. Moreover, metformin was effective at inhibiting OxLDL-induced prostatic proliferation, suggesting the therapeutic potential of this drug in treating BPH in dyslipidemic conditions, with this observation deserving further research. Finally, this study also demonstrated that stromal cells release extracellular vesicles in response to OxLDL, which had not been previously reported for prostatic stromal cells in BPH. These EVs promote the proliferation of both stromal and prostatic epithelial cells, indicating an important role in cell communication and proliferation within the prostate environment and opening new avenues for future research on their content and function. Together, these findings highlight the relationship between dyslipidemia and BPH progression, emphasizing the potential of metformin and targeting EVs for future therapeutic strategies.

## Data Availability

The original contributions presented in this study are included in the article and its Supplementary Material. Further inquiries can be directed to the corresponding author.

## Supplementary Materials

The following supporting information can be downloaded at https://www.mdpi.com/article/10.3390/biology13100827/s1: Figure S1: Morphology of HPSCs from primary cultures derived from patients with BPH; Figure S2: OxLDL is internalized by THP-1 cells, but it does not induce changes in cell morphology or ultrastructure.

## References

[1] Cannarella R., Condorelli R.A., Barbagallo F., La Vignera S., Calogero A.E. (2021). Endocrinology of the Aging Prostate: Current Concepts. Front. Endocrinol..

[2] Vuichoud C., Loughlin K.R. (2015). Benign prostatic hyperplasia: Epidemiology, economics and evaluation. Can. J. Urol..

[3] Smith R.D., Patel A. (2011). Transurethral resection of the prostate revisited and updated. Curr. Opin. Urol..

[4] Gutiérrez-Fisac J.L., Royo-Bordonada M.Á., Rodríguez-Artalejo F. (2006). Riesgos asociados a la dieta occidental y al sedentarismo: La epidemia de obesidad. Gac. Sanit..

[5] Reiner Ž. (2017). Hypertriglyceridaemia and risk of coronary artery disease. Nat. Rev. Cardiol..

[6] Virani S.S., Alonso A., Benjamin E.J., Bittencourt M.S., Callaway C.W., Carson A.P., Chamberlain A.M., Chang A.R., Cheng S., Delling F.N. (2020). Heart Disease and Stroke Statistics-2020 Update: A Report From the American Heart Association. Circulation.

[7] Sebastianelli A., Gacci M. (2018). Current Status of the Relationship Between Metabolic Syndrome and Lower Urinary Tract Symptoms. Eur. Urol. Focus..

[8] Gacci M., Corona G., Vignozzi L., Salvi M., Serni S., De Nunzio C., Tubaro A., Oelke M., Carini M., Maggi M. (2015). Metabolic syndrome and benign prostatic enlargement: A systematic review and meta-analysis. BJU Int..

[9] Carvajal-Carvajal C. (2015). LDL oxidada y la aterosclerosis. Med. Leg Costa Rica.

[10] Yu S., Wong S.L., Lau C.W., Huang Y., Yu C.M. (2011). Oxidized LDL at low concentration promotes in-vitro angiogenesis and activates nitric oxide synthase through PI3K/Akt/eNOS pathway in human coronary artery endothelial cells. Biochem. Biophys. Res. Commun..

[11] Zhang C., Adamos C., Oh M.J., Baruah J., Ayee M.A., Mehta D., Wary K.K., Levitan I. (2017). oxLDL induces endothelial cell proliferation via Rho/ROCK/Akt/p27kip1 signaling: Opposite effects of oxLDL and cholesterol loading. Am. J. Physiol.-Cell Physiol..

[12] Galle J., Hansen-Hagge T., Wanner C., Seibold S. (2006). Impact of oxidized low density lipoprotein on vascular cells. Atherosclerosis.

[13] Seasholtz T.M., Zhang T., Morissette M.R., Howes A.L., Yang A.H., Brown J.H. (2001). Increased expression and activity of RhoA are associated with increased DNA synthesis and reduced p27(Kip1) expression in the vasculature of hypertensive rats. Circ. Res..

[14] Vignozzi L., Rastrelli G., Corona G., Gacci M., Forti G., Maggi M. (2014). Benign prostatic hyperplasia: A new metabolic disease?. J. Endocrinol. Investig..

[15] Van Niel G., Carter D.R.F., Clayton A., Lambert D.W., Raposo G., Vader P. (2022). Challenges and directions in studying cell-cell communication by extracellular vesicles. Nat. Rev. Mol. Cell Biol..

[16] Bruno S., Grange C., Deregibus M.C., Calogero R.A., Saviozzi S., Collino F., Morando L., Falda M., Bussolati B., Tetta C. (2009). Mesenchymal stem cell-derived microvesicles protect against acute tubular injury. J. Am. Soc. Nephrol..

[17] Hergenreider E., Heydt S., Tréguer K., Boettger T., Horrevoets A.J., Zeiher A.M., Scheffer M.P., Frangakis A.S., Yin X., Mayr M. (2012). Atheroprotective communication between endothelial cells and smooth muscle cells through miRNAs. Nat. Cell Biol..

[18] Ding J., Zhang Y., Cai X., Zhang Y., Yan S., Wang J., Zhang S., Yin T., Yang C., Yang J. (2021). Extracellular vesicles derived from M1 macrophages deliver miR-146a-5p and miR-146b-5p to suppress trophoblast migration and invasion by targeting TRAF6 in recurrent spontaneous abortion. Theranostics.

[19] Dong L., Pu Y., Zhang L., Qi Q., Xu L., Li W., Wei C., Wang X., Zhou S., Zhu J. (2018). Human umbilical cord mesenchymal stem cell-derived extracellular vesicles promote lung adenocarcinoma growth by transferring miR-410. Cell Death Dis..

[20] He S., Wu C., Xiao J., Li D., Sun Z., Li M. (2018). Endothelial extracellular vesicles modulate the macrophage phenotype: Potential implications in atherosclerosis. Scand. J. Immunol..

[21] Nguyen M.A., Karunakaran D., Geoffrion M., Cheng H.S., Tandoc K., Perisic Matic L., Hedin U., Maegdefessel L., Fish J.E., Rayner K.J. (2018). Extracellular Vesicles Secreted by Atherogenic Macrophages Transfer MicroRNA to Inhibit Cell Migration. Arterioscler. Thromb. Vasc. Biol..

[22] Wang H., Wang Z.H., Kong J., Yang M.Y., Jiang G.H., Wang X.P., Zhong M., Zhang Y., Deng J.T., Zhang W. (2012). Oxidized low-density lipoprotein-dependent platelet-derived microvesicles trigger procoagulant effects and amplify oxidative stress. Mol. Med..

[23] Roldán Gallardo F.F., Quintar A.A. (2021). The pathological growth of the prostate gland in atherogenic contexts. Exp. Gerontol..

[24] Gómez-Elías M.D., Rainero Cáceres T.S., Giaccagli M.M., Guazzone V.A., Dalton G.N., De Siervi A., Cuasnicu P.S., Cohen D.J., Da Ros V.G. (2019). Association between high-fat diet feeding and male fertility in high reproductive performance mice. Sci. Rep..

[25] Peinetti N., Rubio M.M.C., Sosa L.D.V., Scalerandi M.V., Alasino R.V., Peyret V., Nicola J.P., Beltramo D.M., Quintar A.A., Maldonado C.A. (2020). Testosterone-loaded GM1 micelles targeted to the intracellular androgen receptor for the specific induction of genomic androgen signaling. Int. J. Pharm..

[26] Zago V., Sanguinetti S., Brites F., Berg G., Verona J., Basilio F., Wikinski R., Schreier L. (2004). Impaired high density lipoprotein antioxidant activity in healthy postmenopausal women. Atherosclerosis.

[27] Théry C., Amigorena S., Raposo G., Clayton A. (2006). Isolation and Characterization of Exosomes from Cell Culture Supernatants and Biological Fluids. Curr. Protoc. Cell Biol..

[28] Welsh J.A., Goberdhan D.C., O’Driscoll L., Buzas E.I., Blenkiron C., Bussolati B., Cai H., Di Vizio D., Driedonks T.A., Erdbrügger U. (2024). Minimal information for studies of extracellular vesicles (MISEV2023): From basic to advanced approaches. J. Extracell. Vesicles.

[29] Peinetti N., Scalerandi M.V., Cuello Rubio M.M., Leimgruber C., Nicola J.P., Torres A.I., Quintar A.A., Maldonado C.A. (2018). The Response of Prostate Smooth Muscle Cells to Testosterone Is Determined by the Subcellular Distribution of the Androgen Receptor. Endocrinology.

[30] Oregel-Cortez M.I., Frayde-Gómez H., Quintana-González G., García-González V., Vazquez-Jimenez J.G., Galindo-Hernández O. (2023). Resistin Induces Migration and Invasion in PC3 Prostate Cancer Cells: Role of Extracellular Vesicles. Life.

[31] Luo F., Guo Y., Ruan G.Y., Long J.K., Zheng X.L., Xia Q., Zhao S.P., Peng D.Q., Fang Z.F., Li X.P. (2017). Combined use of metformin and atorvastatin attenuates atherosclerosis in rabbits fed a high-cholesterol diet. Sci. Rep..

[32] Wang Z.S., Huang H.R., Zhang L.Y., Kim S., He Y., Li D.L., Farischon C., Zhang K., Zheng X., Du Z.Y. (2017). Mechanistic study of inhibitory effects of metformin and atorvastatin in combination on prostate cancer cells in vitro and in vivo. Biol. Pharm. Bull..

[33] Pytlowanciv E.Z., Ribeiro D.L., Tamarindo G.H., Taboga S.R., Góes R.M. (2022). High-fat diet during sexual maturation induces hyperplastic differentiation of rat prostate and higher expression of AR45 isoform and ERα. Reprod. Biol..

[34] Galle J., Heinloth A., Wanner C., Heermeier K. (2001). Dual effect of oxidized LDL on cell cycle in human endothelial cells through oxidative stress. Kidney Int..

[35] Seibold S., Schürle D., Heinloth A., Wolf G., Wagner M., Galle J. (2004). Oxidized LDL induces proliferation and hypertrophy in human umbilical vein endothelial cells via regulation of p27Kip1 expression: Role of RhoA. J. Am. Soc. Nephrol..

[36] González-Chavarría I., Fernandez E., Gutierrez N., González-Horta E.E., Sandoval F., Cifuentes P., Castillo C., Cerro R., Sanchez O., Toledo J.R. (2018). LOX-1 activation by oxLDL triggers an epithelial mesenchymal transition and promotes tumorigenic potential in prostate cancer cells. Cancer Lett..

[37] Haga N., Akaihata H., Hata J., Hiraki H., Honda R., Tanji R., Onagi A., Koguchi T., Hoshi S., Ogawa S. (2019). The association between local arteriosclerosis of the prostatic arteries and chronic inflammation in human benign prostatic enlargement. Prostate.

[38] Van Haastert P.J.M. (2020). Unified control of amoeboid pseudopod extension in multiple organisms by branched F-actin in the front and parallel F-actin/myosin in the cortex. PLoS ONE.

[39] Yang J., Liu X., Wang W., Chen Y., Liu J., Zhang Z., Wu C., Jiang X., Liang Y., Zhang J. (2022). Bioelectric fields coordinate wound contraction and re-epithelialization process to accelerate wound healing via promoting myofibroblast transformation. Bioelectrochemistry.

[40] Barron D.A., Rowley D.R. (2012). The reactive stroma microenvironment and prostate cancer progression. Endocr. Relat. Cancer.

[41] Villa M., Cerda-Opazo P., Jimenez-Gallegos D., Garrido-Moreno V., Chiong M., Quest A.F., Toledo J., Garcia L. (2020). Pro-fibrotic effect of oxidized LDL in cardiac myofibroblasts. Biochem. Biophys. Res. Commun..

[42] Vignozzi L., Gacci M., Cellai I., Santi R., Corona G., Morelli A., Rastrelli G., Comeglio P., Sebastanelli A., Maneschi E. (2013). Fat boosts, while androgen receptor activation counteracts, BPH-associated prostate inflammation. Prostate.

[43] Morale M.G., Tamura R.E., Rubio I.G.S. (2022). Metformin and Cancer Hallmarks: Molecular Mechanisms in Thyroid, Prostate and Head and Neck Cancer Models. Biomolecules.

[44] Deza Z., Caimi G.R., Noelia M., Coli L., Ridruejo E., Alvarez L. (2022). Atorvastatin shows antitumor effect in hepatocellular carcinoma development by inhibiting angiogenesis via TGF-β1/pERK signaling pathway. Mol. Carcinog..

[45] Zhu Z., Cao Y., Liu L., Zhao Z., Yin H., Wang H. (2022). Atorvastatin regulates the migration and invasion of prostate cancer through the epithelial-mesenchymal transformation and matrix metalloproteinase pathways. Investig. Clin. Urol..

[46] Saraei P., Asadi I., Kakar M.A., Moradi-Kor N. (2019). The beneficial effects of metformin on cancer prevention and therapy: A comprehensive review of recent advances. Cancer Manag. Res..

[47] Maaninka K., Neuvonen M., Kerkelä E., Hyvärinen K., Palviainen M., Kamali-Moghaddam M., Federico A., Greco D., Laitinen S., Öörni K. (2023). OxLDL sensitizes platelets for increased formation of extracellular vesicles capable of finetuning macrophage gene expression. Eur. J. Cell Biol..

[48] Wang X., Xu F., Kou H., Zheng Y., Yang J., Xu Z., Fang Y., Sun W., Zhu S., Jiang Q. (2023). Stromal cell-derived small extracellular vesicles enhance radioresistance of prostate cancer cells via interleukin-8-induced autophagy. J. Extracell. Vesicles.

[49] Aalberts M., Stout T.A.E., Stoorvogel W. (2014). Prostasomes: Extracellular vesicles from the prostate. Reproduction.

[50] Liu Y., Li Q., Hosen M.R., Zietzer A., Flender A., Levermann P., Schmitz T., Frühwald D., Goody P., Nickenig G. (2019). Atherosclerotic Conditions Promote the Packaging of Functional MicroRNA-92a-3p Into Endothelial Microvesicles. Circ. Res..

[51] Vlaeminck-Guillem V. (2018). Extracellular vesicles in prostate cancer carcinogenesis, diagnosis, and management. Front. Oncol..

[52] Shephard A.P., Giles P., Mbengue M., Alraies A., Spary L.K., Kynaston H., Gurney M.J., Falcón-Pérez J.M., Royo F., Tabi Z. (2021). Stroma-derived extracellular vesicle mRNA signatures inform histological nature of prostate cancer. J. Extracell. Vesicles.

[53] Agarwal V., Yadav S.S., Kumar S., Mehta N., Talwar G., Qadri J., Sarwar S. (2022). Evaluating the role of extracellular vesicles as a biomarker under transmission electron microscope in prostate cancer and benign prostate hyperplasia patients. Urología.

