# Supplementary material for: Extracellular Vesicles Contribute to Oxidized LDL-Induced Stromal Cell Proliferation in Benign Prostatic Hyperplasia
Source: Biology (Basel). 2024 Oct 16;13(10):827. doi: 10.3390/biology13100827 (PMC11504470; doi:10.3390/biology13100827)
Supplement: Supplementary file 1 [file biology-13-00827-s001.zip › Supplementary Figure Legends.pdf]

Supplementary Figure S1: Morphology of HPSCs from primary cultures derived from patients with BPH. Cellular morphology of HPSCs shows a phenotype similar to that of a fibroblast with the characteristic spindle-shaped morphology with the nucleus located in the central zone: A) HPSCs stained with Toluidine Blue, highlighting the general morphology. B) HPSCs visualized using a fluorescence microscope with Phalloidin labeling for cytoplasmic actin filaments (red) and DAPI for the cell nucleus (blue). C) HPSCs imaged with differential interference contrast (DIC) microscopy illustrating cell shape and structure. D) HPSCs observed by transmission electron microscopy (TEM) corroborates the morphology observed in the previous images, providing detailed ultrastructural information.

Supplementary Figure S2: OxLDL is internalized by THP-1 cells, but it does not induce changes in cell morphology or ultrastructure. A) Internalization of OxLDL molecules (yellow arrows) by THP-1 cells observed by TEM. B) Ultrastructure of THP-1 cells evaluated by TEM, showing no morphological changes after treatment with OxLDL1 at 20µg/mL for 24 hours. Scale bars: 5µm. C) The organelle count for THP-1 cells, comparing control vs. OxLDL1 treatment, did not show statistically significant differences for * $p < 0.05$. The test was performed in two replicates, each in triplicate.
