# Supplementary figures and images for: Extracellular Vesicles Contribute to Oxidized LDL-Induced Stromal Cell Proliferation in Benign Prostatic Hyperplasia
Source: Biology (Basel). 2024 Oct 16;13(10):827. doi: 10.3390/biology13100827 (PMC11504470; doi:10.3390/biology13100827)

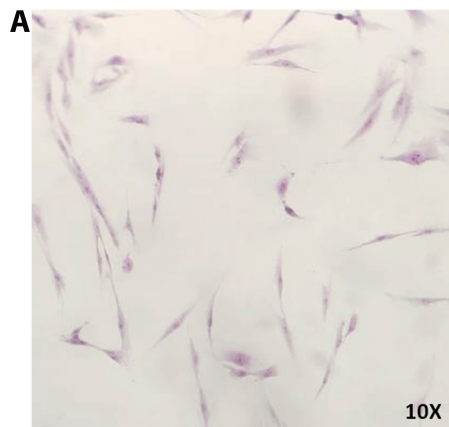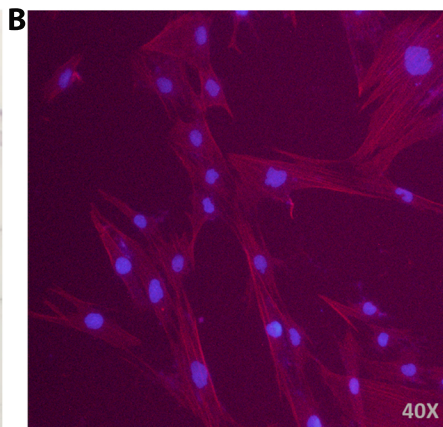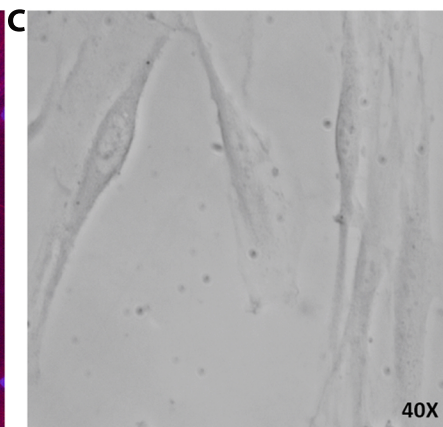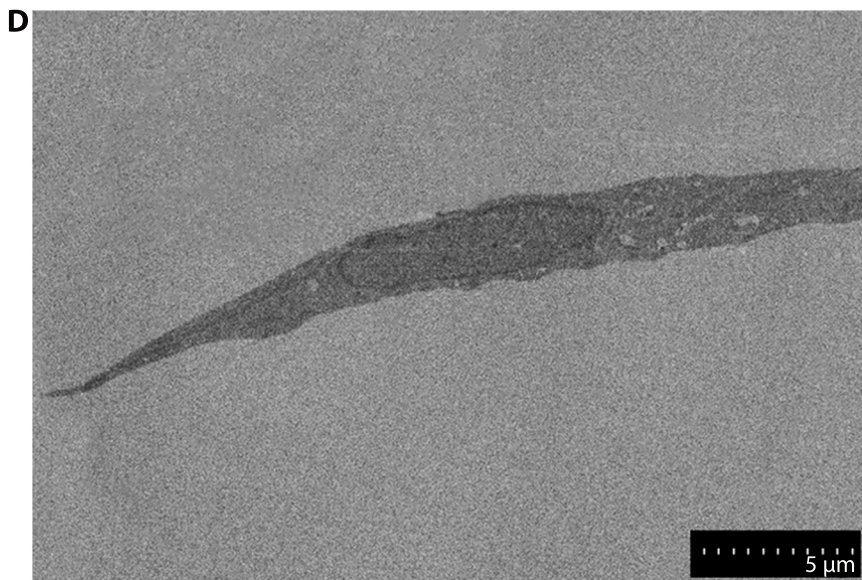

Supplement: Supplementary file 1 [file biology-13-00827-s001.zip › Supplementary Figure S1.pdf]

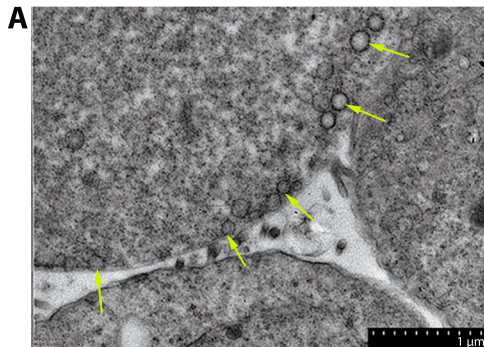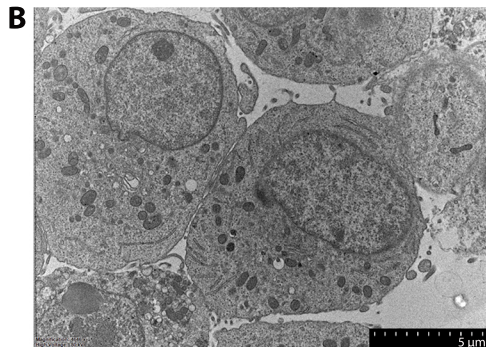

Control

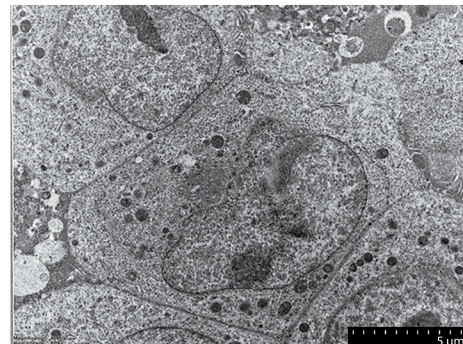

OxLDL 20  $\mu$ g/mL

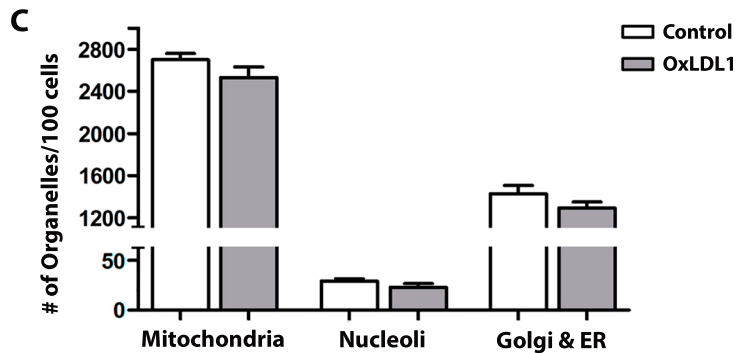

Supplement: Supplementary file 1 [file biology-13-00827-s001.zip › Supplementary Figure S2.pdf]
